# Supplementary material for: Brain-specific homeobox Bsx specifies identity of pineal gland between serially homologous photoreceptive organs in zebrafish
Source: Commun Biol. 2019 Oct 7;2:364. doi: 10.1038/s42003-019-0613-1 (PMC6779754; doi:10.1038/s42003-019-0613-1)
Supplement: Supplementary file 1 — Supplementary Information [file 42003_2019_613_MOESM1_ESM.pdf]

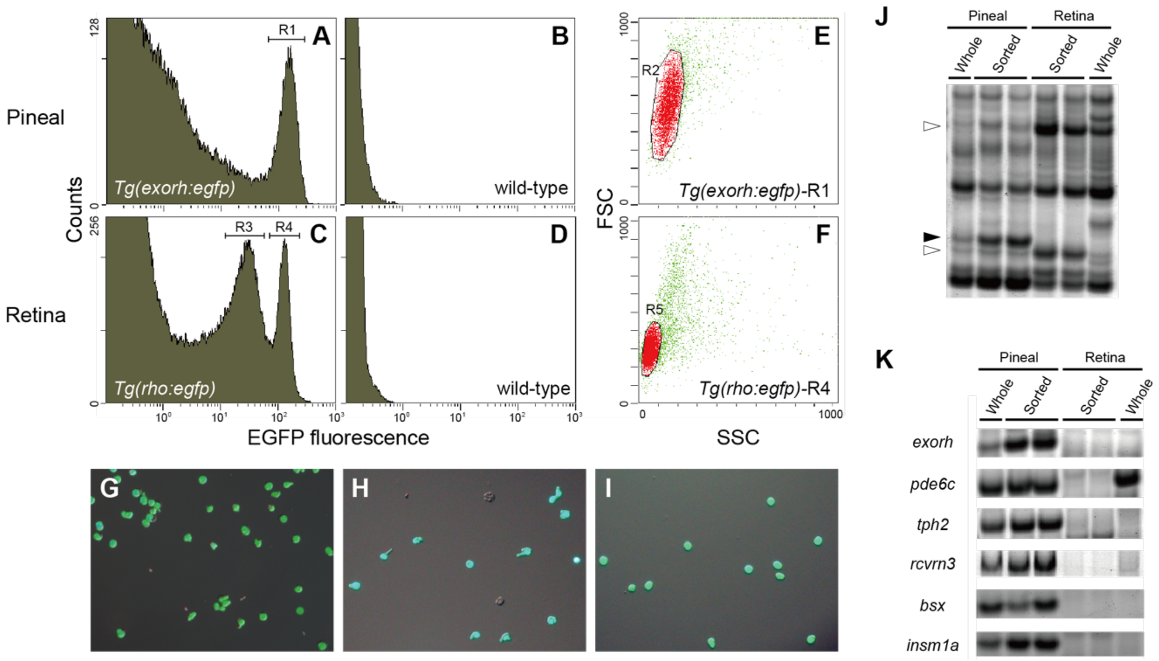

Supplementary Figure 1. Identification of transcription factors expressed selectively in the zebrafish pineal gland. (*A-I*) Sorting of retinal rod and pineal photoreceptor cells by fluorescence-activated cell sorting. (*A-D*) Sorting profiles of pineal (*A* and *B*) and retinal (*C* and *D*) cells prepared from *Tg(exorh:egfp)* hemizygous (*A*), *Tg (rho:egfp)* hemizygous (*C*) and wild-type (*B* and *D*) fish. (*E* and *F*) Scattering profiles of cells gated by R1 (*A*) or R4 (*C*) regions. (G) Sorted pineal cells gated by R2 region in (*E*). Nomarski and fluorescent images are merged. (*H* and *I*) Sorted retinal cells gated by R3 (*H*) or R4 (*I*) regions in (*C*). Many cells in R3 fraction had highly irregular shapes (*H*), whereas those in R4 fraction exhibited homogenous round shapes (*I*). The dual-peak distribution in (*C*) possibly reflects the separation of rod outer segments (R3 fraction) from cell bodies (R4 fraction), which occurred during the preparation of single cell suspension. Pineal photoreceptor cells (R1) in R2 region and retinal rod photoreceptor neurons (R4) in R5 region were used for subsequent ordered-differential-display analysis. (*J*) A representative image of ordered-differential-display gels. In addition to duplicated pools of sorted cells, whole organ/tissue samples were analyzed to validate the cell purification mediated by fluorescence-activated cell sorting. Solid and open arrowheads indicate ordered-differential-display bands enriched in pineal and retinal photoreceptor cells, respectively. Among approximately 7,000 gene fragments detected by the ordered-differential-display analysis, 264 fragments were specific to or highly enriched in pineal photoreceptor cells. (*K*) Examples of pineal-enriched genes detected in ordered-differential-display analysis. A cone photoreceptor-specific gene, *pde6c,* was detected in sorted pineal cells and the whole retina, but not in sorted retinal cells, suggesting the enrichment of retinal rod photoreceptor cells. Full images of the electrophoreses (*J, K*) are shown in Supplementary Fig. 8. FSC, forward scatter; SSC, side scatter.

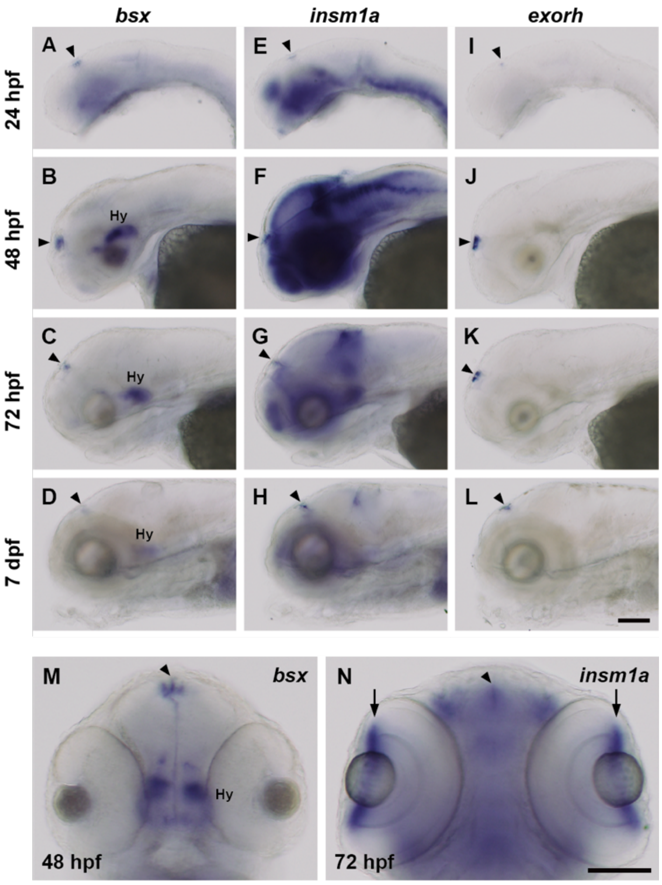

Supplementary Figure 2. Spatial and temporal expression patterns of zebrafish *bsx* and *insm1a*. (*A*-*L*) Expression patterns of *bsx* (*A*-*D*), *insm1a* (*E*-*H*) and *exorh* (*I*-*L*) mRNAs in the zebrafish embryos and larvae. All embryos and larvae are viewed laterally, with anterior to the left and dorsal up. Arrowheads indicate the position of the pineal gland. (*M*) Frontal view of the embryo in (*B*) with dorsal up. (*N*) Dorsal view of the embryo in (*G*) with anterior to the top. Arrows indicate *insm1a* expression in the ciliary marginal zone of the retina. Scale bars = 100 μm. Hy, hypothalamus.

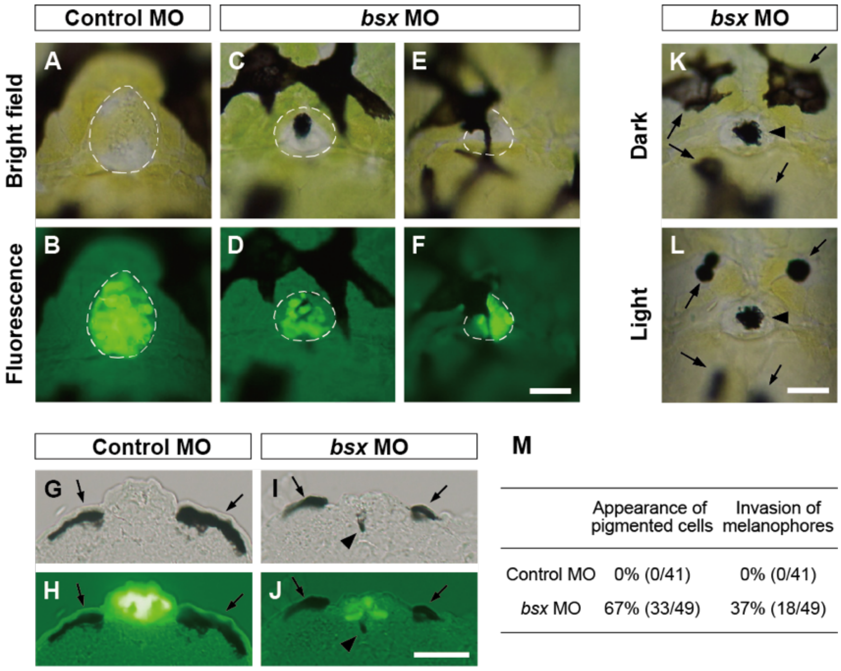

	Appearance of pigmented cells	Invasion of melanophores
Control MO	0% (0/41)	0% (0/41)
bsx MO	67% (33/49)	37% (18/49)

Supplementary Figure 3. Abnormalities of pigmented cells in the pineal gland of *bsx* morphant. *Tg(exorh:egfp)* larvae were used for clear visualization of the pineal gland. (*A*-*F*) Bright field images (*A*, *C* and *E*) and GFP fluorescence images under weak background illumination (*B*, *D* and *F*) of a control larva (*A* and *B*) or *bsx* MO-injected larvae (*C*-*F*) at 6.5 dpf. Bsx depletion led to the emergence of pigmented cells within the pineal gland (*C* and *D*) and the invasion of dermal melanophores onto the dorsal surface of the pineal gland (*E* and *F*). All larvae are viewed dorsally with anterior to the top. White dashed lines indicate the pineal gland. (*G*-*J*) Cross sections of control MO- or *bsx* MO-injected larvae of *Tg(exorh:egfp)* at 6.5 dpf. Bright field images (*G* and *I*) and GFP fluorescence images under weak background illumination (*H* and *J*) are shown with dorsal up. Arrows indicate dermal melanophores, while arrowheads indicate pigmented cell(s) emerged in the pineal gland of *bsx* morphant. (*K* and *L*) Light-induced response of pigmented cells. Unlike dermal melanophores (indicated by arrows), pineal pigmented cells induced by Bsx depletion (indicated by arrowheads) showed no pigment aggregation under light conditions. (*M*) Frequencies of the pigmented cell-related phenotypes. Numbers of larvae with phenotypic abnormalities were counted at any time points of 3.5, 6.5 and 9.5 dpf. The difference between *bsx* MO-injected and control groups is statistically significant for each phenotype ($P=1.1x10^{-12}$; two-sided, Fisher's exact test). Scale bars = 30 μm.

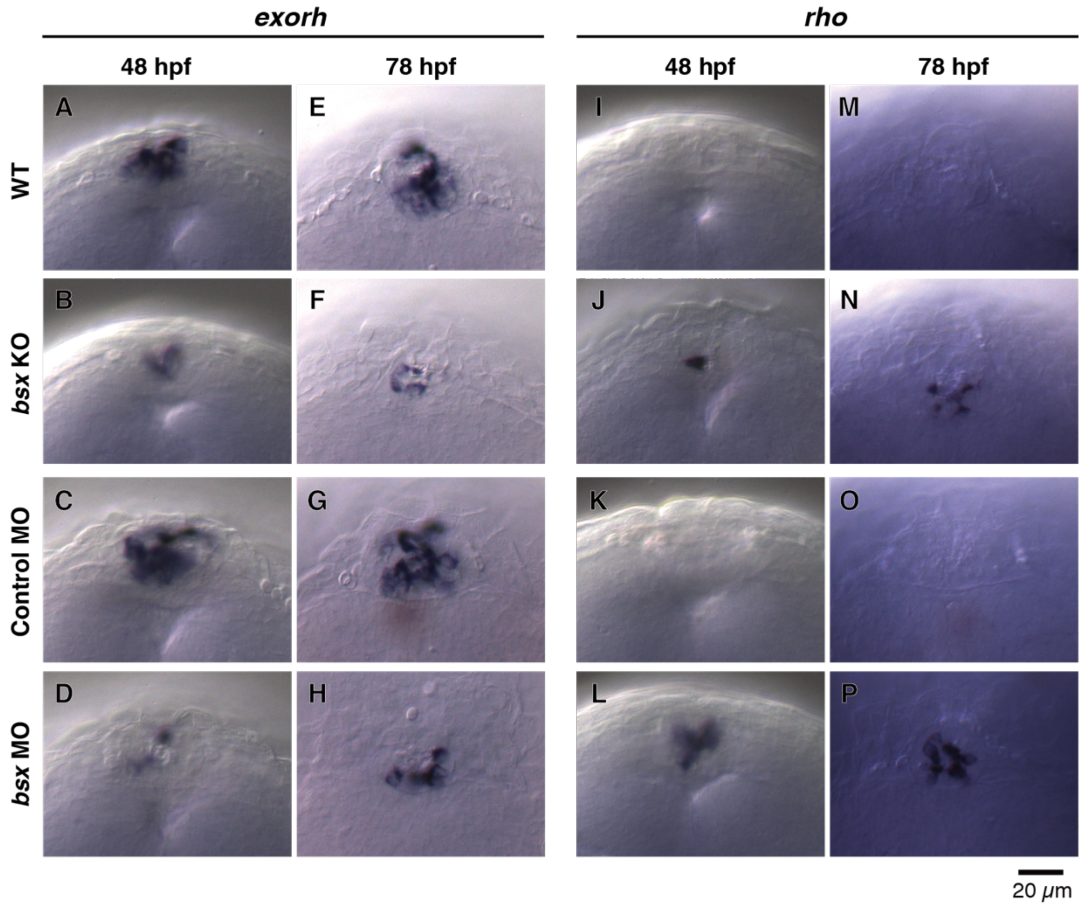

Supplementary Figure 4. Whole-mount *in situ* hybridization of *bsx*^{m1376} (*bsx* KO), wild-type (WT), *bsx* MO-injected (*bsx* MO) and control MO-injected (Control MO) embryos. *exorh* (*A-H*) or *rho* (*I-P*) expression in the pineal gland was examined at 48 and 78 hpf. Embryos were viewed frontally with dorsal to the top at 48 hpf (*A-D, I-L*) or dorsally with anterior to the top at 78 hpf (*E-H, M-P*).

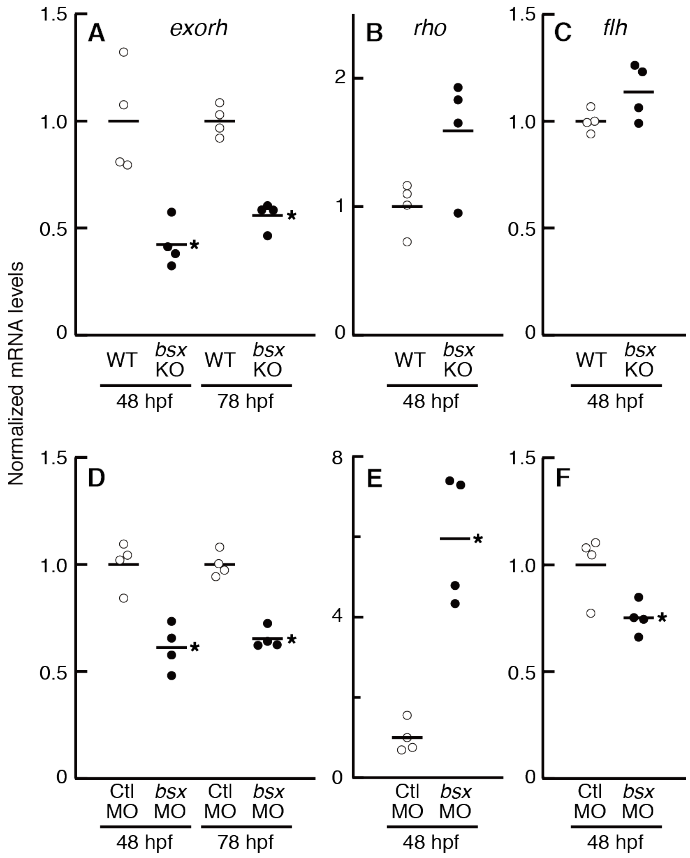

Supplementary Figure 5. Relative mRNA levels of *exorh*, *rho* and *flh* in the anterior segments of 48- or 78-hpf embryos. The mRNA levels in *bsx*^{m1376} embryos (*bsx* KO) were compared to wild-type (WT) (*A-C*), and those in *bsx* MO-injected ones (*bsx* MO) to control MO-injected (Ctl MO) (*D-F*). The *rho* and *flh* mRNA levels were examined only at 48 hpf. In each sample prepared from six (48-hpf) or four (78-hpf) embryos as a replicate, the mRNA levels were measured by quantitative PCR and normalized to the *actb2* mRNA level. Horizontal bars indicate mean values of normalized mRNA levels (n = 4). *P=0.013 (WT/*bsx* KO 48 hpf, A), P=0.00011 (WT/*bsx* KO 78 hpf, A), P=0.0024 (Ctl MO/*bsx* MO 48 hpf, D), P=0.00013 (Ctl MO/*bsx* MO 78 hpf, D), P=0.0069 (Ctl MO/*bsx* MO 48 hpf, E), P=0.039 (Ctl MO/*bsx* MO 48 hpf, F) by Welch's two-sided *t*-test.

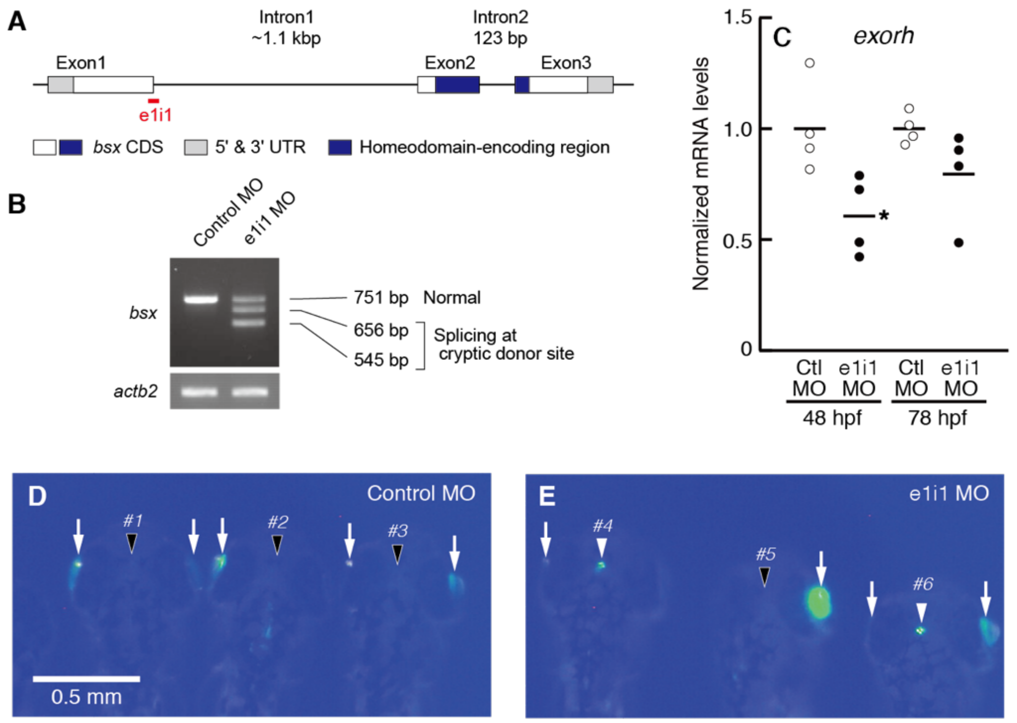

Supplementary Figure 6. *bsx* knock-down mediated by e1i1 MO. (*A*) Schematic illustration of the target site of e1i1 MO. (*B*) RT-PCR of *bsx* mRNA in 48-hpf embryos injected with control or e1i1 MO. Full images of the electrophoreses are shown in Supplementary Fig. 8. (*C*) Relative mRNA levels of *exorh* in the anterior segments of e1i1 MO-injected embryos (e1i1 MO) were compared to control MO-injected ones (Ctl MO) at 48 and 78 hpf. In each sample prepared from six embryos as a replicate, the mRNA levels were measured by quantitative PCR and normalized to the *actb2* mRNA level. Horizontal bars indicate mean values of normalized mRNA levels (n = 4). *P=0.029 by two-sided Welch's *t*-test (Ctl MO/e1i1 MO 48 hpf). (*D, E*) Induction of EGFP expression in the pineal gland of *Tg(rho:egfp)* by e1i1 MO injection. The pineal EGFP induction (*white arrowheads*) was detected at 6.5 dpf in some of the e1i1-injected larvae (#4 and #6, *E*). None of the control MO-injected embryos (#1-#3, *D*) showed EGFP signal in the pineal gland (*black arrowheads*). The ocular fluorescence signals detected through the lens were indicated by arrows.

position	1	2	3	4	5	6	7	8	9	10	11	12	13	14	15	16	17	18
	C	A	C	G	G	T	A	A	T	C	G	G	T	C	T	G	C	C
	C	A	G	G	T	T	A	A	T	T	G	C	T	T	G	G	T	G
	A	A	C	G	T	T	A	A	T	C	G	T	T	T	G	G	G	G
	G	C	A	G	T	T	A	A	T	C	G	T	T	T	G	G	T	G
	G	C	C	G	C	T	A	A	T	C	G	T	T	T	G	G	G	G
	C	A	C	G	G	T	A	A	T	C	G	G	T	T	A	G	C	C
	C	T	G	G	C	T	A	A	T	T	A	G	T	G	G	G	C	C
	G	C	C	A	C	T	A	A	T	C	G	T	T	C	T	T	G	G
	C	A	C	G	C	T	A	A	T	C	G	G	T	G	G	A	G	T
	G	G	C	G	T	T	A	A	T	T	G	G	T	T	G	G	G	C
	C	G	T	G	T	T	A	A	T	G	G	G	C	G	A	G	T	C
	T	A	C	G	A	T	A	A	T	G	G	G	T	T	C	T	G	G
	C	G	C	G	A	T	A	A	T	G	G	G	T	T	G	A	T	T
SAAB1	A	C	C	G	T	T	A	A	T	G	G	G	T	G	G	G	T	G
	C	A	C	A	T	T	A	A	T	C	G	G	T	G	C	T	G	G
	G	G	G	C	T	T	A	A	T	C	G	G	T	G	G	A	G	T
	G	G	C	T	T	T	A	A	T	C	G	T	T	C	G	T	G	G
	G	T	C	G	C	T	A	A	T	C	G	G	T	T	T	G	G	C
	C	A	C	G	C	T	A	A	T	C	G	G	T	T	G	G	G	G
	C	G	G	G	C	T	A	A	T	G	G	G	C	C	G	G	T	A
	G	G	T	G	G	T	A	A	T	C	G	G	T	G	G	A	G	T
	C	A	C	G	T	T	A	A	T	C	G	G	T	G	C	C	G	A
	G	G	C	G	G	T	A	A	T	T	G	G	C	C	T	C	G	T
	G	G	C	G	T	T	A	A	T	C	G	G	T	G	G	A	G	T
	G	G	T	G	T	T	A	A	T	T	G	G	C	G	A	G	T	C
	G	G	C	G	G	T	A	A	T	T	A	G	T	G	C	G	C	C
	C	G	G	G	C	T	A	A	T	C	G	G	T	T	A	C	A	G
	T	G	T	A	G	T	A	A	T	C	G	G	T	G	G	A	G	T
	G	G	C	G	G	T	A	A	T	C	G	A	T	G	C	C	C	C
	T	G	C	G	C	T	A	A	T	C	G	C	C	C	G	A	G	T
	G	C	G	A	C	T	A	A	T	C	G	G	T	T	T	G	G	G
	G	C	C	G	G	T	A	A	T	C	G	G	C	G	A	G	T	C
	T	C	C	C	C	T	A	A	T	C	G	G	T	C	G	A	C	T
	G	T	T	G	C	T	A	A	T	C	G	G	T	T	T	G	G	G
	C	C	T	C	C	T	A	A	T	T	G	G	C	T	G	G	A	G
	G	C	A	G	C	T	A	A	T	G	G	G	T	G	G	A	G	T
	C	G	T	G	C	T	A	A	T	T	G	G	T	T	G	G	T	G
	A	A	G	C	C	T	A	A	T	G	G	G	C	C	G	A	C	A
	C	C	C	G	A	T	A	A	T	G	G	G	T	G	T	G	G	A
	T	G	T	G	G	T	A	A	T	C	G	G	T	G	G	A	G	T
SAAB2	G	A	G	C	C	T	A	A	T	T	G	G	T	G	T	G	G	A
	C	G	G	C	C	T	A	A	T	C	G	G	T	G	T	C	C	A
	G	G	C	T	G	T	A	A	T	T	G	G	C	C	T	G	T	G
	T	G	T	G	C	T	A	A	T	G	G	C	C	C	G	A	G	T
	C	A	G	C	T	T	A	A	T	C	G	G	G	G	G	A	G	T
	G	G	C	G	C	T	A	A	T	G	G	G	A	T	G	T	G	G
	T	G	C	G	T	T	A	A	T	C	G	G	T	G	G	A	G	T
	C	G	G	G	G	T	A	A	T	C	G	G	G	G	G	A	G	T
	T	G	T	G	G	T	A	A	T	T	G	G	C	G	A	G	T	C
	G	T	C	G	T	T	A	A	C	C	T	G	T	T	G	G	G	G
	T	G	A	T	T	T	A	A	T	C	G	G	T	G	G	A	G	T
	A	C	T	G	C	T	A	A	T	C	G	G	T	G	G	C	C	G
	T	G	T	G	G	T	A	A	T	C	G	G	T	G	G	A	G	T
	G	G	G	G	C	T	A	A	T	C	G	A	T	G	T	C	C	G
	C	G	C	G	G	T	A	A	T	C	G	G	T	A	G	C	G	G
	G	G	C	G	C	T	A	A	T	G	G	G	T	T	G	C	T	C
	T	G	T	G	C	T	A	A	T	C	G	G	T	G	G	A	G	T
	A	G	T	G	C	T	A	A	T	C	G	G	T	G	G	A	G	T
	G	G	C	C	T	T	A	A	T	C	G	A	T	G	G	G	G	G
	A	C	T	G	C	T	A	A	T	C	G	G	C	C	G	A	G	T
	G	A	C	G	C	T	A	A	T	C	G	G	T	G	G	G	A	G
	C	G	G	G	C	T	A	A	T	G	G	G	T	T	A	A	T	G
	G	G	G	G	C	T	A	A	T	T	G	G	T	T	G	G	A	G
	T	G	A	G	G	T	A	A	T	T	G	G	T	G	G	A	G	T
	G	G	C	G	G	T	A	A	T	C	G	G	T	C	C	T	G	G
	G	C	C	G	G	T	A	A	T	C	G	G	T	C	T	G	T	G
SAAB3	C	A	C	G	A	T	A	A	T	C	G	G	T	C	C	G	C	G
	T	G	C	G	C	T	A	A	T	C	G	G	T	G	G	A	G	T
	G	A	G	C	G	T	A	A	T	T	G	G	C	C	G	G	A	G
	T	G	C	G	T	T	A	A	T	G	G	G	C	G	A	G	T	C
	C	G	G	C	C	T	A	A	T	C	G	G	T	G	C	A	C	G
	G	A	A	G	C	T	A	A	T	C	G	G	C	C	C	G	A	C
	G	C	C	C	C	T	A	A	T	C	G	G	T	G	G	A	G	T
	C	G	C	G	G	T	A	A	T	C	G	G	T	T	G	C	T	T
	G	C	C	G	C	T	A	A	T	C	G	G	T	G	G	A	G	T
	G	G	G	G	C	T	A	A	T	C	G	G	T	T	G	G	G	A
	G	G	T	G	T	T	A	A	T	C	G	G	T	G	G	A	G	T
	G	C	C	T	C	T	A	A	T	C	G	G	T	G	T	T	G	G
	G	C	C	G	C	T	A	A	T	T	G	C	T	T	T	G	C	G
A	6	16	5	4	4	0	79	79	0	0	2	3	1	1	8	27	6	7
T	14	4	16	4	19	79	0	0	78	15	1	5	61	23	14	7	16	26
G	36	42	17	60	19	0	0	0	0	13	76	67	2	39	48	35	44	32
C	23	17	41	11	37	0	0	0	1	51	0	4	15	16	9	10	13	14
consensus	G	G	C	G	C	**T**	**A**	**A**	**T**	**C**	**G**	**G**	**T**	G	G	G	G	G

Supplementary Figure 7. Alignment of Bsx binding sequences obtained by SAAB. A total of 79 sequences were obtained from three independent SAAB experiments (SAAB1, 2 and 3). A position-specific score matrix and a consensus sequence are shown at the bottom. Nucleotides identical to the consensus sequence are highlighted with gray background.

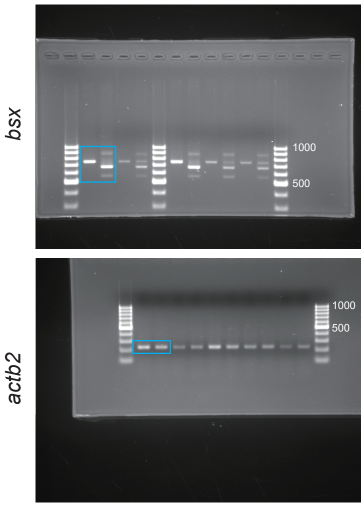

Figure 1B

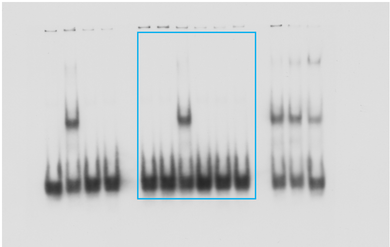

Figure 5B

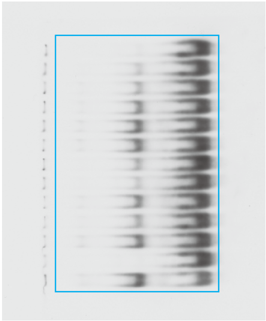

Figure 5D

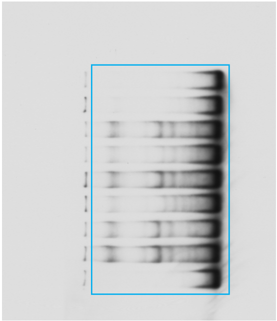

Figure 5E

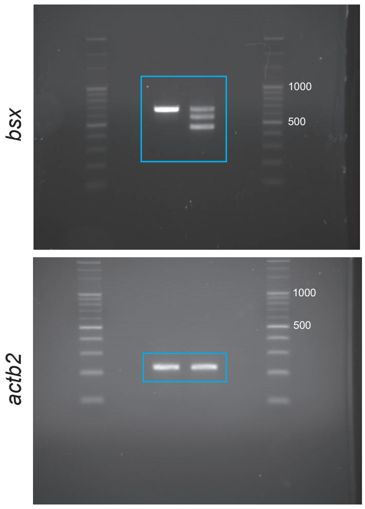

Supplementary Figure 6B

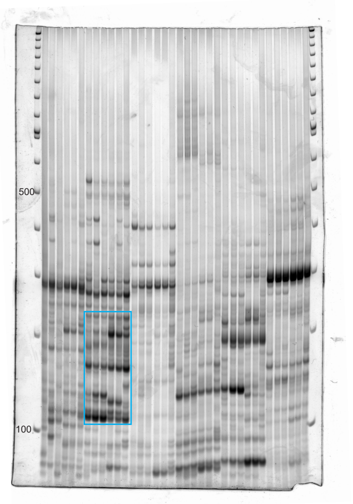

Supplementary Figure 1J

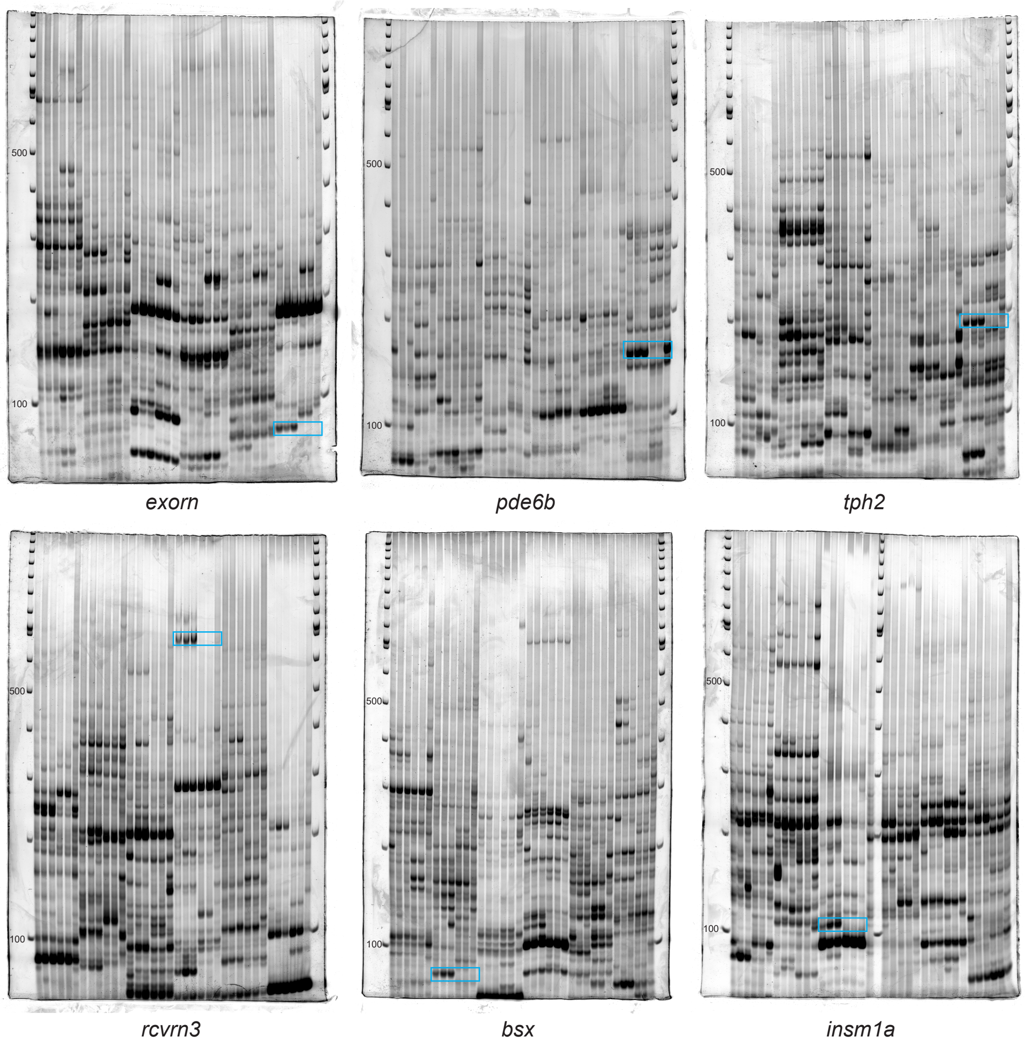

Supplementary Figure 1K

Supplementary Figure 8. Full images of all the gel data shown in the manuscript.

Supplementary Table 1.
Potential recognition sequences of Bsx in promoter/enhancer regions of pineal-specific genes.

promoter/enhancer*	sequence#	start	end	ori$	matching score
zebrafish *aanat2* (-1650/+123)	TCTAATAGATGT	-415	-404	F	6.95
	GTTAATTGAGGA	-1595	-1584	F	6.80
	GTTAATTGTACC	-357	-346	F	6.53
	ACTAATTGAAGT	-1609	-1598	F	6.38
zebrafish *aanat2* PRDM (+1207/+1463)	GATAATCTGATC	1410	1421	F	6.72
seabream *aanat2* (-1352/+237)	GCTAATTGCTGT	-262	-251	F	7.86
	CCTAATCTGTCA	-458	-447	F	7.18
	ACTAATTTGTAG	-761	-750	F	6.95
	TTTAATTAGTCT	-679	-668	F	6.49
chicken *pinopsin* (-1554/-1)	CTTAATGGGAAC	-650	-661	R	6.48
	TGTAATGCGTTC	-1270	-1259	F	6.47
	CCTAATCCCTCA	-689	-700	R	6.37
	TTTAATCGAGTA	-216	-227	R	6.34
rat *PINA* (-238/-1)	GCTAATTAGTAA	-40	-29	F	7.16
	GCTAATCTGGCT	-49	-38	F	7.13
	GGTAATTGCGTG	-16	-5	F	7.11
	TGTAATTAGGGG	-107	-96	F	6.47
	AGTAATCTGAGT	-199	-188	F	6.47
zebrafish *bsx* (-948/+53)	GCTAATCTACTT	-70	-59	F	6.57

#Sequences with an intact TAAT core and high matching scores (>6.20; calculated using the score matrix in Fig. 5*A*) were extracted from zebrafish *aanat2* promoter and its downstream enhancer PRDM[1], seabream *aanat2* promoter[2], chicken *pinopsin* promoter[3], rat *PINA* promoter[4] and zebrafish *bsx* promoter.

$The orientation of sequences relative to the direction of transcription; the same (F) or reverse (R) direction.

*No sequence that perfectly matches the consensus binding sequence of Bsx (TAATCGGT) was found in any of these promoters or in the *exorh* promoter (Fig. 5). This is possibly due to CpG methylation *in vivo*, which may impair the binding affinity of Bsx.

References

1 Appelbaum, L. *et al.* Zebrafish serotonin-*N*-acetyltransferase-2 gene regulation: pineal-restrictive downstream module contains a functional E-box and three photoreceptor conserved elements. *Mol Endocrinol* **18**, 1210-1221 (2004).

2 Zilberman-Peled, B. *et al.* Transcriptional regulation of arylalkylamine-*N*-acetyltransferase-2 gene in the pineal gland of the gilthead seabream. *J Neuroendocrinol* **19**, 46-53 (2007).

3 Takanaka, Y., Okano, T., Yamamoto, K. & Fukada, Y. A negative regulatory element required for light-dependent *pinopsin* gene expression. *J Neurosci* **22**, 4357-4363 (2002).

4 Li, X. *et al.* A pineal regulatory element (PIRE) mediates transactivation by the pineal/retina-specific transcription factor CRX. *Proc Natl Acad Sci U S A* **95**, 1876-1881 (1998).
