# Supplementary material for: Brain-specific homeobox Bsx specifies identity of pineal gland between serially homologous photoreceptive organs in zebrafish
Source: Commun Biol. 2019 Oct 7;2:364. doi: 10.1038/s42003-019-0613-1 (PMC6779754; doi:10.1038/s42003-019-0613-1)
Supplement: Supplementary file 2 — Reporting Summary [file 42003_2019_613_MOESM2_ESM.pdf]

nature research

Corresponding author(s): Daisuke Kojima & Yoshitaka Fukada

Last updated by author(s): Sep 14, 2019

Reporting Summary

Nature Research wishes to improve the reproducibility of the work that we publish. This form provides structure for consistency and transparency in reporting. For further information on Nature Research policies, see Authors & Referees and the Editorial Policy Checklist.

Statistics

For all statistical analyses, confirm that the following items are present in the figure legend, table legend, main text, or Methods section.

n/a	Confirmed	
☐	☒	The exact sample size (*n*) for each experimental group/condition, given as a discrete number and unit of measurement
☐	☒	A statement on whether measurements were taken from distinct samples or whether the same sample was measured repeatedly
☐	☒	The statistical test(s) used AND whether they are one- or two-sided<br>*Only common tests should be described solely by name; describe more complex techniques in the Methods section.*
☒	☐	A description of all covariates tested
☐	☒	A description of any assumptions or corrections, such as tests of normality and adjustment for multiple comparisons
☐	☒	A full description of the statistical parameters including central tendency (e.g. means) or other basic estimates (e.g. regression coefficient) AND variation (e.g. standard deviation) or associated estimates of uncertainty (e.g. confidence intervals)
☒	☐	For null hypothesis testing, the test statistic (e.g. *F*, *t*, *r*) with confidence intervals, effect sizes, degrees of freedom and *P* value noted<br>*Give P values as exact values whenever suitable.*
☒	☐	For Bayesian analysis, information on the choice of priors and Markov chain Monte Carlo settings
☒	☐	For hierarchical and complex designs, identification of the appropriate level for tests and full reporting of outcomes
☒	☐	Estimates of effect sizes (e.g. Cohen's *d*, Pearson's *r*), indicating how they were calculated

Our web collection on statistics for biologists contains articles on many of the points above.

Software and code

Policy information about availability of computer code

Data collection	No specific software was used for data collection.
Data analysis	We used R for statistical analyses, Image J v1 for the quantification of images, and Microsoft Excel for calculating the SAAB-matrix matching scores.

For manuscripts utilizing custom algorithms or software that are central to the research but not yet described in published literature, software must be made available to editors/reviewers. We strongly encourage code deposition in a community repository (e.g. GitHub). See the Nature Research guidelines for submitting code & software for further information.

Data

Policy information about availability of data

All manuscripts must include a data availability statement. This statement should provide the following information, where applicable:

- Accession codes, unique identifiers, or web links for publicly available datasets
- A list of figures that have associated raw data
- A description of any restrictions on data availability

Data available on request from the authors.

Field-specific reporting

Please select the one below that is the best fit for your research. If you are not sure, read the appropriate sections before making your selection.

☒ Life sciences ☐ Behavioural & social sciences ☐ Ecological, evolutionary & environmental sciences

Life sciences study design

All studies must disclose on these points even when the disclosure is negative.

Sample size	Sample size was determined as N ≥ 4 for animal experiments and N = 3 for others.
Data exclusions	No data were excluded from the analyses.
Replication	We verified the reproducibility of the experimental finding.
Randomization	Allocation in this study is random.
Blinding	n/a

Reporting for specific materials, systems and methods

We require information from authors about some types of materials, experimental systems and methods used in many studies. Here, indicate whether each material, system or method listed is relevant to your study. If you are not sure if a list item applies to your research, read the appropriate section before selecting a response.

Materials & experimental systems

n/a	Involved in the study	
☒	☐	Antibodies
☐	☒	Eukaryotic cell lines
☒	☐	Palaeontology
☐	☒	Animals and other organisms
☒	☐	Human research participants
☒	☐	Clinical data

Methods

n/a	Involved in the study	
☒	☐	ChIP-seq
☐	☒	Flow cytometry
☒	☐	MRI-based neuroimaging

Eukaryotic cell lines

Policy information about cell lines

Cell line source(s)	The HEK293T cell line used in this study has the same origin as our previous papers, such as Torii et al. (FEBS Lett 581:5327-31, 2007) and Asada et al. (J Neurosci 27:11769-75, 2007).
Authentication	The cell line was not authenticated.
Mycoplasma contamination	We confirmed that the cell line was negative for mycoplasma.
Commonly misidentified lines (See ICLAC register)	n/a

Animals and other organisms

Policy information about studies involving animals; ARRIVE guidelines recommended for reporting animal research

Laboratory animals	RIKEN WT, EkkWill and ABTL were used as wild-type strains of zebrafish (Danio rerio). The ages of the animals used in this study were described in the figure legends of the manuscript. In general, information on sex is not available at the embryonic stages of zebrafish.
Wild animals	n/a
Field-collected samples	n/a
Ethics oversight	All the animal experiments were conducted according to the animal ethics guideline of the University of Tokyo.

Note that full information on the approval of the study protocol must also be provided in the manuscript.

Flow Cytometry

Plots

Confirm that:

☒ The axis labels state the marker and fluorochrome used (e.g. CD4-FITC).

☒ The axis scales are clearly visible. Include numbers along axes only for bottom left plot of group (a 'group' is an analysis of identical markers).

☒ All plots are contour plots with outliers or pseudocolor plots.

☒ A numerical value for number of cells or percentage (with statistics) is provided.

Methodology

Sample preparation	All the procedure of sample preparation was described in the Materials and methods section of manuscript.
Instrument	EPICS ELITE ESP cytometer (Beckman Coulter)
Software	Expo32 software (Beckman Coulter)
Cell population abundance	We confirmed that almost all cells in the post sorted fractions were GFP-positive by visual inspection using an aliquot. The total numbers of sorted cells were 106,203 and 124,955 for Tg(exorh:egfp) and Tg(rho:egfp) , respectively.
Gating strategy	Forward scatter, side scatter and 525-nm green fluorescence intensity were monitored for gating.

☒ Tick this box to confirm that a figure exemplifying the gating strategy is provided in the Supplementary Information.
